# Lack of fitness costs associated with resistance to permethrin in *Musca domestica*

**DOI:** 10.1038/s41598-023-50469-7

**Published:** 2024-01-02

**Authors:** Hafiz Azhar Ali Khan

**Affiliations:** Institute of Zoology, University of the Punjab, Lahore, Pakistan

**Keywords:** Entomology, Zoology, Environmental sciences

## Abstract

Resistance to permethrin has been reported in Pakistani strains of *Musca domestica*. The present study explored the performance of biological traits and analyzed life tables to determine whether there is any detrimental effect of permethrin resistance on the fitness of permethrin-resistant strains [an isogenic resistant strain (Perm-R) and a field strain (Perm-F)] compared to a susceptible strain (Perm-S). Perm-R and Perm-F exhibited 233.93- and 6.87-fold resistance to permethrin, respectively. Life table analyses revealed that the Perm-R strain had a significantly shorter preadult duration, longer longevity, shorter preoviposition period, higher fecundity, finite rate of increase, intrinsic rate of increase, net reproductive rate and a shorter mean generation time, followed by the Perm-F strain when compared to the Perm-S strain. Data of the performance of biological traits reveled that permethrin resistance strains had a better fit than that of the Perm-S strain. The enhanced fitness of resistant strains of *M. domestica* may accelerate resistance development to permethrin and other pyrethroids in Pakistan. Some possible measures to manage *M. domestica* and permethrin resistance in situations of fitness advantage are discussed.

## Introduction

*Musca domestica* Linnaeus (the common house fly) is a hazardous pest all over the globe and plays an active role in the mechanical transmission of pathogens of dysentery, cholera, salmonellosis, and trachoma^1–3^. *M. domestica* has the ability to transmit *Yersinia pseudotuberculosis* (Pfeiffer), a gram-negative bacteria, in poultry birds, leading to their death^4^. Every year, *M. domestica* infestations in and/or around dairy facilities, livestock farms and urban environments result in huge economic losses in the form of disease transmissions, lowered dairy and livestock production and massive capital investment in the management of *M. domestica* and treatment of diseased animals^2,5,6^. The major factors that encourage their spread and associated problems are poor sanitary conditions and the evolution of resistance to insecticide classes, including pyrethroids^7–12^.

The use of permethrin, a pyrethroid, is an integral part for controlling insect pests of animals, poultry birds and urban environments all over the world might be due to its relatively low mammalian toxicity and high efficacy against insect pests^13^. Permethrin is a broad-spectrum insecticide that has been proved effective against several insect pests^14^, including *M. domestica*^15^. Pyrethroid insecticides act on protein of voltage-gated sodium channel and interfere in the normal activity of the pore channel that leads to repetitive nerve firing, paralysis and ultimately mortality of exposed insects. Frequent permethrin applications in pest management programs have resulted in the wide-spread development of resistance in several insects^16–19^, including *M. domestica*^20,21^. Increased expression of metabolic detoxification enzymes such as glutathione transferase or cytochrome P450s, and target site insensitivity have been found major mechanisms responsible for permethrin resistance^22^.

Several factors affect the development of resistance against insecticides. For instance, frequency of insecticide applications, inheritance mode, frequency of resistance alleles, insects’ biology, population dynamics of insects, and weak performance of biological traits (i.e., fitness costs)^23,24^. The performance of biological traits of resistant strains is likely to be varied between species, insecticides, and species with different geographical origins^25^. Resistant insects usually show fitness costs (e.g., lengthened development period, reduced fecundity and survival etc.) probably because of a resource and energy reallocation at the cost of development and metabolic mechanisms and hence are less fit in their environment^26^.

If the fitness cost happens in a species as a result of insecticide resistance development, it will limit an increase in resistance alleles and resistant insects will restore their susceptibility when we uplift selection pressure by temporarily discontinuing certain insecticide(s) or rotation with alternate insecticides that do not exhibit cross-resistance^27,28^. Therefore, for the successful management of insect pests and insecticide resistance, it is of utmost importance to have better understanding regarding insecticide resistance, cross-resistance and performance of biological traits of resistant insects. Previously, resistance to permethrin and cross-resistance in *M. domestica* have been reported from Punjab, Pakistan^7,29^. However, information about the fitness of biological traits of *M. domestica* is lacking. The goal of the present study was to study biological traits and analyze life tables to determine if there is any difference in fitness of permethrin resistant strains compared to a susceptible strain. The data can be helpful to further understand the impact of permethrin resistance under field conditions, and to develop a resistance management program.

## Materials and methods

### *Musca domestica* strains

Three strains of *M. domestica* were used to study their biological parameters: Perm-S, Perm-F and Perm-R. The Perm-S was a permethrin susceptible/reference strain, collected from an urban area of Lahore (31.5546° N, 74.3572° E) and reared in the laboratory for > 2 years without exposure to insecticides. The Perm-F strain (unselected) was a relatively permethrin resistant strain^30^, compared with the Perm-S strain, collected from a dairy farm in Lahore. The Perm-R was a permethrin resistant near-isogenic line of *M. domestica*^29^, which was developed from the Perm-F strain. All strains were maintained in the entomological laboratory [26 ± 2 °C, 65 ± 5% relative humidity (RH), 12:12 h light/dark photoperiod] by a well-established methodology^31,32^.

### Bioassays

Technical-grade permethrin (98.9%; Chem Service Inc. West Chester, PA, USA) was used to evaluate its toxicity against the Perm-S strain. Topical bioassays were conducted following the methodology of Liu and Yue^20^ and Khan et al.^33^. Permethrin was dissolved in acetone to seven concentrations that caused < 100% and > 0% mortality, and the range of the concentrations in bioassays was 1.5 to 100 ng/fly. The insecticide solution of a specific concentration was applied on the thoracic notum (0.5 µL/fly) of 3–5-day-old female *M. domestica* using a micropipette (0.1–2 μL, Acura ® manual 825, Socorex, Switzerland). All the concentrations were replicated on three separate times by preparing fresh solutions. There were 20 *M. domestica* exposed against each concentration per replicate. *M. domestica* received topical application of acetone alone in control bioassays. After exposure to permethrin concentrations or acetone, treated *M. domestica* were shifted into plastic jars of 250 mL capacity. In order to avoid food shortage during bioassays, 20% sugar solution was provided via cotton wicks into each jar. Mortality data observations were made after 48 h of exposure to permethrin concentrations or acetone.

### Comparison of biological traits and population parameters of Perm-S, Perm-F and Perm-R strains

Biological traits and population parameters of *M. domestica* strains were studied following the methodology described in our previous reports^34,35^. In order to harvest fresh eggs, ten pairs of adult male and female *M. domestica* (less than 1-day-old) of Perm-S, Perm-F or Perm-R were kept separately in wooden-screen cages (23 × 23 × 37 cm). Each cage was consisted of powdered-milk, sugar and water to feed adult *M. domestica*, and an egg laying substrate (“a paste of sugar, powdered milk, yeast, wheat straw and wheat bran at a ratio of 3:3:10:20:40 by weight, respectively, mixed with 250 mL of water”) in Petri plates. This substrate also acts as a larval diet of *M. domestica*^36^. Fifty freshly laid eggs from each strain cage were taken and shifted gently into ten glass beakers (five eggs per beaker) containing 75 g larval diet. Beakers were daily observed to record survival rate, longevity, and developmental time from eggs to adults’ eclosion. Emerged adults were paired separately into small cages as stated above, and fecundity and longevity were noted daily until death of each pair.

### Statistical analyses

Mortality data of female flies of the Perm-S strain exposed to different concentrations of permethrin were analyzed to calculate median lethal dose (LD_50_) and LD_95_ values, and associated statistics following Probit protocol^37^, using PoloPlus 2.0v. Data analyses were performed as outlined in our previous report^34^:

“analyses of population parameters (Table 1) and biological traits of different strains of *M. domestica* were performed by the TWOSEX-MSChart program^38,39^. The significance of mean values of population parameters and biological traits of *M. domestica* in different treatments were analyzed through the paired bootstrap test using TWOSEX-MSChart with 100,000 resamplings^40,41^”.

**Table 1:** Equations of population parameters and explanations used in life tables of different strains (Perm-S, Perm-F, Perm-R) of *Musca domestica.*

Parameter	Equation	Definition
Intrinsic rate of increase (*r*)	$\sum_{x=0}^{\infty }{e}^{-r(x+1)}{l}_{x}{m}_{x}=1$	“It is the population growth rate as time approaches infinity and population reaches the stable age-stage distribution. The population size will increase at the rate of *e*^*r*^ per time unit. The Euler-Lotka equation was used to calculate the intrinsic rate of increase with the age indexed from 0”^58^
The finite rate of increase (*λ*)	${\sum }_{n=1}^{\infty }\left({\lambda }^{-\left(x+1\right)}\sum_{j=1}^{m}{f}_{xj}{S}_{xj}\right)=1$	“The finite rate of increase (*λ*) is the population growth rate as time approaches infinity and population reaches the stable age-stage distribution. The population size will increase at the rate of *λ* per time unit”^59^
Net reproductive rate (*R*_*0*_)	$\sum_{x=0}^{\infty }{l}_{x}{m}_{x}= {R}_{0}$	“The total offspring produced by an average individual during its lifetime”^59^
Mean generation time (*T*)	$T= \frac{{{\text{ln}}R}_{0}}{r}$	“The time length that a population increases to *R*_*0*_-fold of its size at stable age-stage distribution”^59^
Age-specific survival rate (*l*_*x*_)	${l}_{x}= \sum_{j=1}^{m}{S}_{xj}$	“Where *m* is the number of stages”^56,59^
Age-specific fecundity (*m*_*x*_)	${m}_{x}=\frac{{\sum }_{j=1}^{m}{S}_{xj}{f}_{xj}}{{\sum }_{j=1}^{m}{S}_{xj}}$	Age-specific fecundity (*m*_*x*_) of the cohort at age *x*^56,59^
Age-stage life expectancy (*e*_*xj*_)	${e}_{xj}=\sum_{i=x}^{\infty }\sum_{y=j}^{m}{S^{\prime}}_{iy}$	*“S’*_*iy*_ is the probability that an individual of age *x* and stage *j* will survive to age *i* and stage *y* by assuming *S*_*xj*_ = 1”^59,60^
Age-stage reproductive value (*v*_*xj*_)	${v}_{xj}=\frac{{e}^{r(x+1)}}{{S}_{xj}} \sum_{i=x}^{\infty }{e}^{-r\left(i+1\right)}\sum_{y=j}^{m}{S^{\prime}}_{iy}{f}_{iy}$	“The contribution of individuals at age *x* and stage *j* to the future population”^59,61–63^

Adapted from Khan^35^ and Iqbal et al.^34^.

### Ethical approval and consent to participate

This article does not describe any studies involving human participants performed by the authors. All applicable international, national and/or institutional guidelines for the care and use of animals were followed.

## Results

### Toxicity of permethrin against Perm-S, Perm-F and Perm-R strains

The Perm-S strain of *M. domestica* was the most susceptible strain to permethrin with the LD_50_ value 14.59 ng/fly, followed by Perm-F and Perm-R strains with LD_50_ values 100.22 and 3413 ng/fly, respectively (Table 2). Perm-F and Perm-R strains were 6.87 and 233.93 fold resistant to permethrin, respectively, in comparison to the Perm-S strain (Table 2).

**Table 2:** Toxicity values and resistance ratios (RR) of Perm-S, Perm-F and Perm-R strains of *Musca domestica*.

Strain	*n*	LD_50_ (95% CI) (ng/fly)	LD_95_ (95% CI) (ng/fly)	Fit of probit line				RR**
				Slope (± SE)	χ^2^	df	*p*	
Perm-S	480	14.59 (10.77–19.98)	105.18 (63.71–133.95)	1.92 (0.15)	8.07	5	0.15	1
Perm-F*	420	100.22 (84.73–118.70)	805.32 (751.21–1005.32)	2.20 (0.19)	2.04	4	0.73	6.87
Perm-R^29^	420	3413 (2447–5267)	38,045 (29,510–44,026)	2.27 (0.23)	8.23	4	0.08	233.93

*Reported previously^7^.

**Resistance ratio: LD_50_ of either Perm-F or Perm-R strain divided by the LD_50_ of the Perm-S strain.

### Life tables of Perm-S, Perm-F and Perm-R strains

In most of the life table parameters, Perm-R and Perm-F strains were significantly the best fit compared to the Perm-S strain (Table 3). Eggs of Perm-R and Perm-F strains hatched in 1.12 ± 0.05 and 1.19 ± 0.06 days, respectively, while eggs of the Perm-S strain took relatively more time to hatch. Larvae of Perm-R and Perm-F strains developed faster (4.36 ± 0.09 and 5.22 ± 0.08 days, respectively) than those of the Perm-S strain (7.27 ± 0.18 days). Pupae of the Perm-R strain took 3.91 ± 0.12 days to convert into the adult stage in comparison to those of Perm-F (4.87 ± 0.13 days) and Perm-S strains (7.27 ± 0.19 days). The total preadult duration of the Perm-R strain was the shortest (9.39 ± 0.40 days), while the Perm-S strain took the longest time to pass through immature stages (16.42 ± 0.26 days). Total longevity of all individuals and longevity of female adult flies of the Perm-R strain was the highest (36.28 ± 2.14 and 42.88 ± 1.62 days, respectively) than the rest of strains. Male adult flies of Perm-R and Perm-F strains survived for 37.56 ± 2.58 and 36.06 ± 3.07 days, respectively, both strains were statistically at par, compared to male flies of the Perm-S strain. However, there was non-significant difference among strains in the case of the proportion of adult females. Female flies of the Perm-S strain took the highest preoviposition time (23.25 ± 0.41 days) than the rest of strains. Female flies of Perm-F and Perm-R strains laid eggs for 8.15 ± 0.70 and 7.85 ± 0.66 days, respectively, while females of the Perm-S laid eggs for a shorter period of time. Similarly, adult female flies of the Perm-R strain laid the highest number of eggs (504.54 ± 15.78 eggs/female), followed by adult females of Perm-F (437.86 ± 31.56 eggs/female) and Perm-S strains (340.35 ± 23.18 eggs/female). Moreover, Perm-R and Perm-F strains exhibited the highest preadult survival rate compared to that of the Perm-S strain (Table 3).

**Table 3:** Comparison of biological traits of permethrin resistant (Perm-R and Perm-F) strain with the permethrin susceptible (Perm-S) of *Musca domestica*.

Biological trait	Perm-S*	Perm-F*	Perm-R*
Egg hatch period (days)	1.68 ± 0.07a	1.19 ± 0.06b	1.12 ± 0.05b
Larval duration (days)	7.27 ± 0.18a	5.22 ± 0.08b	4.36 ± 0.09c
Pupal period (days)	7.27 ± 0.19a	4.87 ± 0.13b	3.91 ± 0.12c
Total preadult duration (days)	16.42 ± 0.26a	11.32 ± 0.28b	9.39 ± 0.40c
Total longevity (all individuals) (days)	20.52 ± 2.10c	28.14 ± 2.21b	36.28 ± 2.14a
Longevity (female) (days)	33.76 ± 1.62b	34.32 ± 2.06b	42.88 ± 1.62a
Longevity (male) (days)	33.56 ± 1.91b	36.06 ± 3.07a	37.56 ± 2.58a
Proportion of adult females *N*_*f*_/*N*	0.34 ± 0.06a	0.44 ± 0.09a	0.52 ± 0.07a
Preoviposition period (TPOP)	23.25 ± 0.41a	16.80 ± 0.33b	12.88 ± 0.32c
Oviposition days (*O*_*d*_)	5.44 ± 0.46b	8.15 ± 0.70a	7.85 ± 0.66a
Fecundity (*F*) (eggs/female)	340.35 ± 23.18c	437.86 ± 31.56b	504.54 ± 15.78a
Preadult survival rate (*S*_*a*_)	0.52 ± 0.07b	0.78 ± 0.10a	0.88 ± 0.07a

*Values are mean ± S.E. (standard error) of biological traits analyzed using the bootstrap technique^39,40^.

The analysis of population parameters of Perm-S, Perm-F and Perm-R strains revealed that the Perm-R strain was at the top on the values of finite rate of increase per day (*λ*), net reproductive rate (*R*_*0*_) and intrinsic rate of increase per day (*r*), followed by Perm-F and Perm-S strains of *M. domestica*. The Perm-R strain exhibited the shortest mean generation time (*T*) (18.03 ± 0.41 days), while the Perm-S strain took 27.18 ± 0.69 days to complete a generation (Table 4).

**Table 4:** Comparison of population increase parameters of permethrin resistant (Perm-R and Perm-F) strain with the permethrin susceptible (Perm-S) of *Musca domestica*.

Parameter	Perm-S*	Perm-F*	Perm-R*
Finite rate of increase (*λ*) (day^−1^)	1.190 ± 0.011c	1.278 ± 0.014b	1.362 ± 0.014a
Intrinsic rate of increase (*r*) (day^−1^)	0.174 ± 0.009c	0.245 ± 0.011b	0.309 ± 0.011a
Net reproductive rate (*R*_*0*_) (offspring)	115.65 ± 24.13c	192.62 ± 33.71b	262.48 ± 36.45a
Mean generation time (*T*) (day)	27.18 ± 0.69a	21.46 ± 0.47b	18.03 ± 0.41c

*Values are mean ± S.E. (standard error) of biological traits analyzed using the bootstrap technique^39,40^.

**Figure 1:**
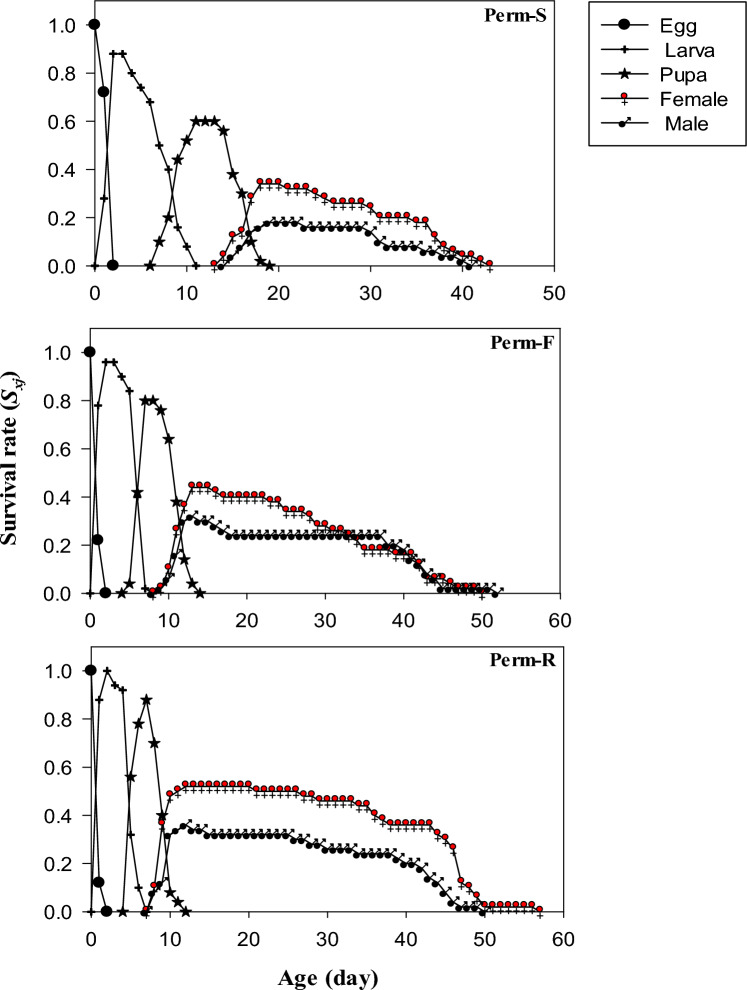
Survival rate (*S*_*xj*_) of permethrin susceptible (Perm-S) and permethrin resistant (Perm-R and Perm-F) strains of *Musca domestica.*

The comparison of the age-stage-specific survival rate (*S*_*xj*_) of individuals of Perm-S, Perm-F and Perm-R strains revealed a significant stage differentiation and stage overlaps, largely due to the differences in the developmental period of flies of the strains (Fig. 1). The survival rate of developmental stages (egg, larva and pupa) was the highest for the Perm-R strain, which was followed by the Perm-F and Perm-S strain. Adult male and female flies of Perm-R and Perm-F strains eclosed earlier as compared to those of the Perm-S strain (Fig. 1).

**Figure 2:**
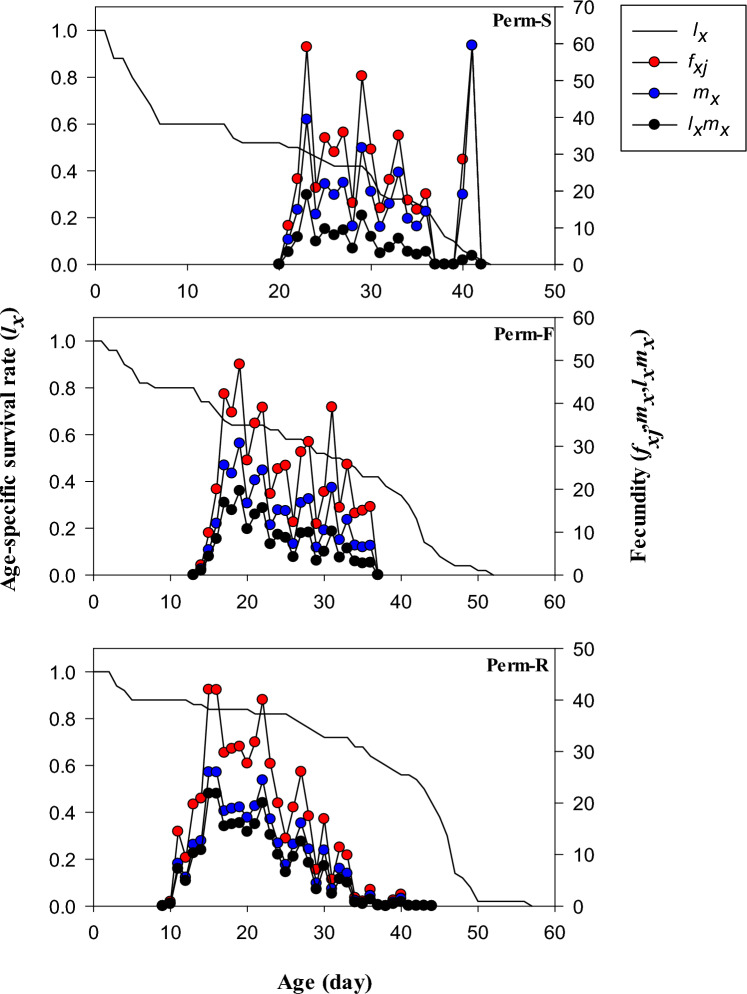
Age-specific survival rate (*l*_*x*_) of permethrin susceptible (Perm-S) and permethrin resistant (Perm-R and Perm-F) strains of *Musca domestica.*

The curves of *f*_*xj*_ and *m*_*x*_ (age-stage-specific fecundity of females, and the total population, respectively) of the Perm-R started to rise at age 09 day as compared to age 13 and 20 days in the case of Perm-F and Perm-S strains, respectively (Fig. 2). Based on the estimated values, the mortality of the last adult fly of Perm-S, Perm-F and Perm-R strains occurred at 42, 52 and 57 days. The *l*_*x*_ curve of the Perm-S strain dropped more rapidly than those of Perm-R and Perm-F strains, indicating a high mortality rate during different time intervals. The curve of *m*_*x*_ (age-specific fecundity) indicated that reproduction of Perm-R, Perm-F and Perm-S strains started at age 9, 13 and 20 days, respectively.

**Figure 3:**
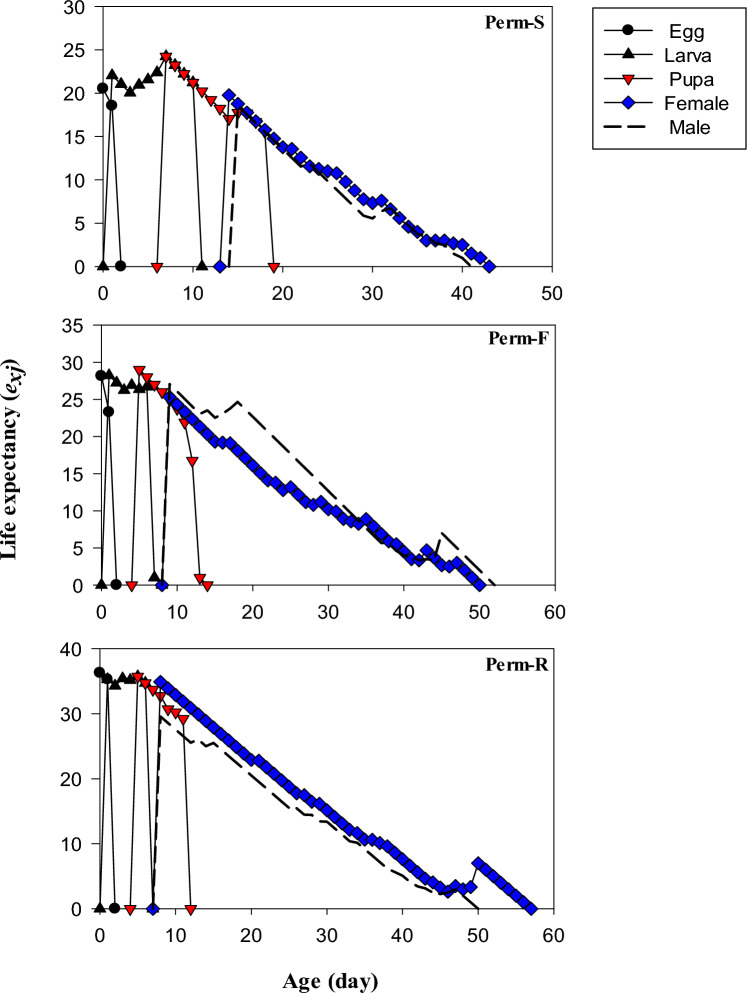
Life expectancy (*e*_*xj*_) of permethrin susceptible (Perm-S) and permethrin resistant (Perm-R and Perm-F) strains of *Musca domestica.*

The life expectancy (*e*_*xj*_) values of Perm-S, Perm-F and Perm-S strains are presented in Fig. 3. The *e*_*xj*_ values of freshly laid eggs of Perm-S, Perm-F and Perm-S strains were 20.52, 28.14 and 36.28 days, respectively, which were exactly the same as those of the total longevity of all individuals in Table 3.

**Figure 4:**
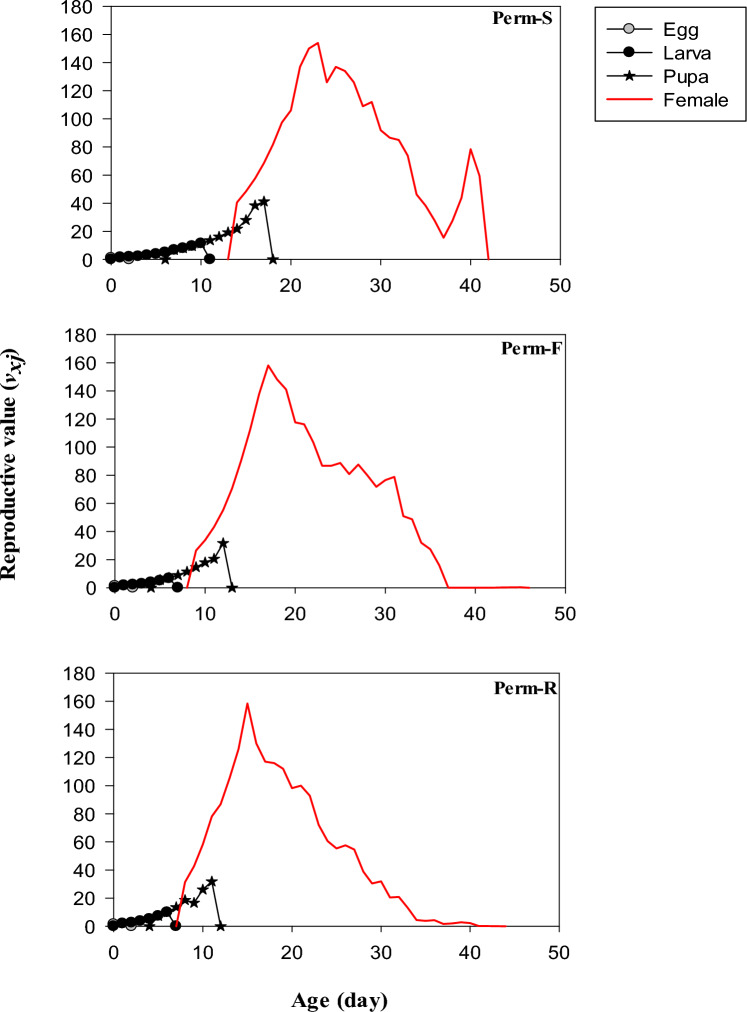
Reproductive value (*v*_*xj*_) of permethrin susceptible (Perm-S) and permethrin resistant (Perm-R and Perm-F) strains of *Musca domestica.*

The reproductive (*v*_*xj*_) values of Perm-S, Perm-F and Perm-S strains are presented in Fig. 4. The *v*_*xj*_ values of freshly oviposited eggs and the finite rate of increase were identical: 1.362, 1.278 and 1.190 per day for Perm-R, Perm-F and Perm-S, respectively. Upon the emergence of adult females, the *v*_*xj*_ value rose to its peak at age 08, 09 and 14 days in Perm-R, Perm-F and Perm-S strains, respectively. When adult female flies began to lay eggs, the *v*_*xj*_ curves for Perm-R, Perm-F and Perm-S strains reached to their highest peaks at age 15, 17 and 23, respectively (Fig. 4).

## Discussion

Variation in fitness of biological traits of individuals in a population helps to detect their genetic contribution and the future projection of populations. Insects face a number of stress factors during their routine activities in the environment. Exposure to agrochemicals is one of the stressors, and insects utilize a lot of energy to make changes in genetic, physiological and behavioral mechanisms to cope with these chemicals, sometimes leading to develop resistance by inducing mutations in the target site or by the production of detoxifying enzymes^26^. Such actions are usually costly and may affect fitness of biological traits of resistant insect pests^27^. However, lack of fitness costs in insecticide resistant insects might be associated with lack of negative pleiotropic effects associated with resistance alleles^42^. If none of the resistance gene has deleterious effects on physiology of resistant insects, insecticide resistance would not be energetically costly, and resistant insect would not show negative deviations in the performance of biological events compared with susceptible strains^24^. In addition, the possibility of the inhibition mechanism of detoxification enzymes^43^ and/or the presence of modifier genes^44^ in insecticide resistant insects can ameliorate their fitness.

The present study revealed that permethrin resistant strains Perm-R and Perm-F took shorter preadult duration, longer longevity, shorter preoviposition period, higher number of oviposition days and fecundity, and high survival rate than those of the permethrin susceptible strain Perm-S. These values resulted in higher finite, intrinsic and net reproductive rates of increase, and shorter mean generation time for Perm-R and Perm-F strains compared with the Perm-S strain. High peak values and the earlier occurrence of *v*_*xj*_ of Perm-R and Perm-F strains revealed that populations of these strains will increase faster than that of the Perm-S strain. In comparison to the Perm-S strain, Perm-F and Perm-R strains exhibited low and high levels of resistance to permethrin, respectively^29,30^. The observed fitness of biological parameters of permethrin resistant strains suggest that permethrin resistance was not energetically costly, and there might be an absence of a trade-off in the distribution of physiological resources to overcome insecticide resistance and to perform biological activities^24^.

Relatively better performance of biological parameters of Perm-R and Perm-F strains than that of the Perm-S strain studied in the present study suggested that there was a lack of fitness cost of resistance to permethrin that could result in the fast development of permethrin resistance. This was also evidenced in our previous report that the field collected strain of *M. domestica* (named ‘Perm-F’ in the present study) rapidly developed very high levels of resistance to permethrin after few rounds of laboratory selections. At the time of collection, Perm-F had the LD_50_ value 100.22 ng/fly for permethrin, and after ten rounds of selection with permethrin the LD_50_ value increased to 3787.08 ng/fly^30^. The permethrin-selected strain was then used to establish a near-isogenic line of *M. domestica* (Perm-R) resistant to permethrin by the backcrossing methodology^29^. A stable nature of permethrin resistance in the above strains in previous reports had postulated the idea of lack of fitness, and the findings of the present work further strengthened the idea of fitness of permethrin resistance in the studied strains.

Performance of biological parameters or fitness cost in insecticide resistant strains of different insects has been evaluated in different studies but with inconsistent findings. For example, Abbas et al.^45^ studied the effects of laboratory selection with imidacloprid insecticide on biological traits of *M. domestica*. Imidacloprid resistance exerted the fitness cost in the selected strain by negatively affecting fecundity, survival rate of different developmental stages, developmental time, generation time, biotic potential and intrinsic rate of increase of *M. domestica*. Variable fitness costs have been reported in *M. domestica* as a result of resistance to pyrethroid insecticides and in the absence of insecticide exposures^46^. Laboratory selection of *M. domestica* with spinosad also resulted in fitness costs of a number of biological events as compared to the susceptible or unselected strains^27^. Basit et al.^24^ reported that laboratory selection of *Bemisia tabaci* (Gennadius) with acetamiprid resulted in lack of fitness costs. The acetamiprid-selected strain exhibited a higher reproductive rate, intrinsic rate, biotic potential than those of the laboratory susceptible and unselected field strains. Recently, Valmorbida et al.^47^ reported that pyrethroid resistant *Aphis glycine* Matsumura represented enhanced reproductive performance and lack of fitness costs of biological events.

In the present study, permethrin resistant strains had a significant fitness advantage (lack of fitness cost) over the susceptible strain. The observed fitness advantage of biological parameters of permethrin resistant strains suggest that permethrin resistance was not energetically costly, and there might be an absence of a trade-off in the distribution of physiological resources to overcome insecticide resistance and to perform biological activities^24^. Previously, in contrast to the present study, it has been reported that pyrethroid insecticides induce fitness costs in insect pests^46,48^. However, studies also revealed that the phenomenon of fitness cost is not a consistent phenomenon; rather it changes from species to species, among insecticides within the same class and/or strains of the same species with different geographical origins. For instance, deltamethrin (a pyrethroid) resistance resulted in a significant fitness cost in a strain of *Aedes albopictus* (Skuse) from China^49^, but exposure to the same insecticide resulted in enhanced fitness in a strain of *Sitophilus zeamais* (Motschulsky) from Brazil^50^. Bifenthrin, another pyrethroid, caused fitness cost in *Rhopalosiphum padi* (L.)^51^; however, Valmorbida et al.^47^ reported lack of fitness cost in *A. glycine* following exposure to bifenthrin. Similarly, exposure to *Bacillus thuringiensis*-based toxins resulted in a significant fitness cost in a strain of *Plutella xylostella* (L.) from Malaysia^52^, but exposure to the same toxin resulted in lack of fitness costs in another strain of *P. xylostella* from Florida^53^. Presence of fitness cost due to a particular insecticide in some species but not in others suggest that there might be a different resistance mechanism in species with fitness cost than those with fitness advantage^54^. Hence, contrasting results of fitness advantage due to pyrethroid resistance in the present study than those reported earlier could be due to the different resistance mechanism and/or due to the differences in geographical origin of the selected strain of *M. domestica*, since environmental conditions could also be important factors in the fitness cost mechanism^15^. The age-stage, two-sex life table theory is an excellent tool to forecast the effects of insecticides on insect populations^55,56^. By this theory, the growth potential of an insect population can be better assessed by studying finite rate of increase (*λ*) as well as intrinsic rate of increase (*r*), both are calculated by using data of biological traits of insect species in question^57^. Both of these calculations indicate the effect of rate of development, survival and fecundity on the fitness of the population under specific conditions. Moreover, these calculations also help to assess an overall impact of resistance allele on resistant insects^24^. Higher values of *λ, r* and *R*_*0*_, and a lower value of *T* for Perm-R strain and Perm-F strain compared to those of the Perm-S strain reflect that permethrin resistance alleles may probably have no detrimental effects on population parameters (Table 4). In this scenario, alleles responsible for developing resistance to insecticides could be maintained in strains after selection pressure since their progenies do not exhibit lack of fitness cost.

## Conclusions

Increased performance of biological traits of permethrin resistant strains is alarming for the success of sustainable management of *M. domestica*. If manifested in the field, certain biological traits of *M. domestica* could evolve resistance to permethrin in heavily treated individuals. Permethrin resistant strains exhibited lack of cross-resistance to propoxur, imidacloprid, profenofos, chlorpyrifos, spinosad and spinetoram^29,30^. In this situation, rotational use of insecticides with different modes of action can possibly be the best option to delay the development of resistance and manage outbreaks of *M. domestica*. Although the data presented here are laboratory-based, increased performance of biological traits of permethrin resistant strains is concerning and require special attention to manage *M. domestica* under the field environment. Further understandings on the distribution of the field evolved resistance to permethrin are required to delay the development of resistance and maintain its efficacy for a longer period of time.

## Data Availability

All data generated or analyzed during this study are included in this published article.
